# Tracking the Fate of Stem Cell Implants with Fluorine-19 MRI

**DOI:** 10.1371/journal.pone.0118544

**Published:** 2015-03-13

**Authors:** Jeffrey M. Gaudet, Emeline J. Ribot, Yuhua Chen, Kyle M. Gilbert, Paula J. Foster

**Affiliations:** 1 Imaging Research Laboratories, Robarts Research Institute, London, ON, Canada; 2 Department of Medical Biophysics, University of Western Ontario, London, ON, Canada; Central Michigan University, UNITED STATES

## Abstract

**Background:**

In this study we used cellular magnetic resonance imaging (MRI) to detect mesenchymal stem cells (MSC) labeled with a Fluorine-19 (^19^F) agent. ^19^F-MRI offers unambiguous detection and in vivo quantification of labeled cells.

**Methods:**

We investigated two common stem cell transplant mouse models: an immune competent, syngeneic transplant model and an immune compromised, xenograft transplant model. ^19^F labelled stem cells were implanted intramuscularly into the hindlimb of healthy mice. The transplant was then monitored for up to 17 days using ^19^F-MRI, after which the tissue was excised for fluorescence microscopy and immunohistochemisty.

**Results:**

Immediately following transplantation, ^19^F-MRI quantification correlated very well with the expected cell number in both models. The 19F signal decreased over time in both models, with a more rapid decrease in the syngeneic model. By endpoint, only 2/7 syngeneic mice had any detectable ^19^F signal. In the xenograft model, all mice had detectable signal at endpoint. Fluorescence microscopy and immunohistochemistry were used to show that the ^19^F signal was related to the presence of bystander labeled macrophages, and not original MSC.

**Conclusions:**

Our results show that ^19^F-MRI is an excellent tool for verifying the delivery of therapeutic cells early after transplantation. However, in certain circumstances the transfer of cellular label to other bystander cells may confuse interpretation of the long-term fate of the transplanted cells.

## Introduction

Stem cell therapy has the potential to play an important role in regenerative medicine. Mesenchymal stromal/stem cells (MSC) have been extensively investigated for clinical application over the past decade.[1,2] MSC are capable of differentiating into a variety of important tissues, such as: bone, cartilage and adipose.[3] They also display immunomodulatory properties.[4–6] Their presence in adult tissue, and ease of expansion *in vitro* has made MSC good candidate cells for clinical translation.[7,8]

In order to advance stem cell therapy, tools must be developed to monitor the survival of implanted stem cells non-invasively after administration to the patient. Magnetic resonance imaging (MRI) cell tracking is an effective method to visualize and monitor cells non-invasively after implantation due to the high spatial resolution and lack of ionizing radiation.

The majority of MRI cell tracking studies have used iron oxide nanoparticles to label the cells of interest.[9–15] When imaged with MRI, the iron nanoparticles produce a dark signal void in T2/T2* weighted proton images. This technique is highly sensitive, permitting the imaging of single cells.[16,17] Limitations with tracking iron-labeled cells arise from low specificity, due to other regions in the image with low signal, and from complicated *in vivo* quantification of the signal loss. Our group and others have shown that degree of signal loss produced by iron labeled cells is only linear at low iron concentrations.[16,18] Furthermore the high sensitivity to iron can produce ambiguity due to the strong false-positive signal produced when even a small number of bystander cells become labeled inadvertantly.[19,20]

As an alternative to iron cell tracking, fluorine-19 (^19^F) MRI with perfluorocarbon (PFC) nanoemulsions has been used for cell tracking.[21] ^19^F MRI is able to image implanted cells with high specificity due to the lack of detectable fluorine in biological tissue.[22,23] Quantification of implanted cells is possible since the ^19^F MRI signal intensity is linearly related to the number of ^19^F-labeled cells. Unlike PET/SPECT probes, ^19^F does not undergo radioactive decay allowing for longitudinal studies without radiation-induced toxicity to the implanted cells or surrounding tissue. Furthermore, the first clinical application of ^19^F-MRI cell tracking for DC immunotherapy was recently reported, showing the technique is both feasible and safe for human application.[24]

In this paper, we investigated the feasibility of quantifying MSC survival in two different immune system environments. This was performed by comparing the change in ^19^F-MRI signal strength over time using two popular transplantation models. A syngeneic transplant model, with mouse MSC (mMSC) implanted in an immune-competent mouse host, was compared to a xenograft model produced from human MSC (hMSC) implanted in an immune-compromised mouse. Our goals were: i) to quantify the apparent cell number non-invasively for 2.5 weeks and ii) to validate *in vivo* ^19^F-MRI quantification results with fluorescence microscopy and immunohistochemistry.

## Methods

### MSC culture and labeling

hMSC came from bone marrow donated by healthy young adult volunteers after written informed consent according to a protocol approved by University Health Network Research Ethics Board (Toronto, Canada)[25]. hMSC were cultured as described by Ribot et al.[19] mMSC derived from the bone marrow of C57Bl/6 mice and expressing green fluorescence protein (GFP+) were purchased from Cyagen Bioscience Inc. (Catalog # MUBMX-01101). The cells were cultured in OriCell Mouse MSC Growth Medium until 90% confluent. The mMSC were passaged once before labeling and implantation.

All MSC were labeled with the red fluorescent perfluoropolyether agent, Cell Sense (CS-ATM-DM Red; CelSense Inc. Pittsburg, USA)[26]. Labeling took place over 24 hours at a concentration of 2.5mg/mL. After incubation, the cells were washed 3 times with Hank’s balanced salt solution (HBSS), harvested with Trypsin, spun down and counted. At this stage the cells were tested for viability using trypan blue exclusion. Intracellular ^19^F content of cells was determined using NMR spectroscopy, as we have described previously.[19]

### MSC implantation

1.5x10^6^ Cell Sense-labeled hMSC in 100μL of HBSS were implanted intramuscularly into the right hindlimb muscle of immune-compromised, nude mice (nu/nu, Charles River Canada) to produce a xenograft model (n = 7). In a similar manner, 2.0x10^6^ labeled mMSC in 100μL of HBSS were implanted into immune competent, C57Bl/6 mice (Jackson Laboratories) producing a syngeneic model (n = 8). In both cases, the injections were performed under 2% isoflurane anesthesia. All experiments involving human and mouse stem cells, as well as animal use, were approved by the Western University Animal Use Committee (AUP 2009–042).

### MRI

All images were collected using a 9.4T Varian small-animal MRI scanner. A 3D balanced steady state free precession (bSSFP) sequence was used for both proton and fluorine imaging. Cell pellets containing 2x10^5^, 4x10^5^, 6x10^5^, 8x10^5^, 1x10^6^ and 2x10^6 19^F-labeled MSC were imaged alongside a reference tube of known fluorine concentration (7.3x10^16 19^F/μL). MRI was performed using a dual-tuned birdcage volume coil (diameter 2.2cm, length 5.1cm), tuned to 400.2 MHz and 376.8 MHz for proton and fluorine imaging respectively. For proton imaging of cell pellets the scan parameters were: repetition time (TR) = 3.8ms, echo time (TE) = 1.9ms, receiver bandwidth (rBW) = 125kHz, flip angle (FA) = 30°, averages = 2, resolution = 200x200x200μm^3^. For fluorine imaging the parameters were: TR = 3.5ms, TE = 1.8ms, rBW = 25kHz, FA = 70°, averages = 250, resolution = 1x1x2mm^3^. Total protocol time for both proton and fluorine imaging was under 90 minutes. The pellets were imaged on three separate occasions to test quantification variability.

Mice containing ^19^F-labeled MSC implantations were imaged at four time points, starting on day 0, after implantation. The scan parameters were the same for the *in vivo* mouse MRI as described previously. During scanning the mice were anesthetized with 2% isoflurane, with breathing rate and temperature monitored. Due to the high sensitivity of bSSFP to off-resonance frequencies[27], a narrow 1.5kHz sinc pulse was used to excite only the ^19^F agent.

### Image analysis and quantification

Prior to image analysis, a signal correction was applied to the ^19^F datasets by subtracting a constant value (x) from all voxels within the dataset using the image program, ImageJ.[28,29] The value of x was equal to the signal of the single voxel containing the lowest signal throughout the entire dataset. This linear translation acted to left-shift the data distribution to begin at zero. Quantification was performed using Voxel Tracker software, as described by Srinivas et al.[30] Briefly, in the ^19^F MR images, the signal contained within a hand drawn ROI is summed and compared to the average signal produced by the reference tube of known concentration (2.6x10^16 19^F spins/μL). This value (X) provides the total number of ^19^F spins located at the ROI. NMR spectroscopy was then performed using a known number of the same transplanted cells, along with a second ^19^F source containing a known number of ^19^F spins. By comparing the relative NMR signals, the number of ^19^F spins per cell (Y) is obtained. Division of X by Y yields the number of cells located at the ROI. Significance between time-points was assessed using a repeated measures, one-way ANOVA.

### Immunohistochemistry

Mice were sacrificed and perfused with 4% paraformaldehyde following the final imaging timepoint. In addition, one mouse from each model was sacrificed and perfused on Day 0 for comparison. The hindlimb muscle was extracted and cryoprotected with increasing concentrations of sucrose (10%, 20%, 30%) before freezing in OCT medium. Tissue was sectioned with a cryostat. Fluorescence microscopy was performed to image the red fluorescent ^19^F-label as well as the GFP+ mMSC with a Zeiss AXIO Imager microscope (Carl Zeiss Canada Ltd). Immunohistochemistry staining was then performed on these sections. Macrophage presence was assessed using Biotin anti-mouse macrophage (F4/80) monoclonal antibody (Cedarlane Laboratories Ltd) with 3,3’-Diaminobenzidine (DAB) counterstain.

## Results

### Labeling of MSC with Cell Sense

**Fig 1:**
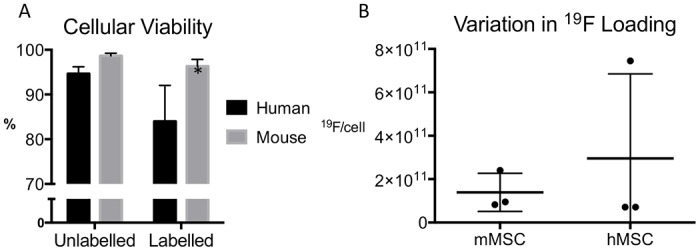
Cellular viability and loading with the ^19^F-agent. (A) Cellular viability was investigated before and after labeling with the ^19^F-agent, Cell Sense. Although a statistically significant difference was observed in hMSC after labeling, the viability remained high (>80%) in all experiments. There was no significant difference in mMSC viability. (B) Cellular loading was determined by performing NMR on a known number of cells alongside a reference peak with a known number of ^19^F atoms. We observed variation in cellular loading of both hMSC and mMSC between experiments. However, this variation does not affect in vivo ^19^F quantification since each transplant was only compared to its specific cellular loading.

Fig. 1 shows that labeling with the ^19^F agent did not negatively affect the mMSC cellular viability. The viability of the hMSC was slightly decreased following labeling. Previous work by our group demonstrated that the perfluorocarbon, Cell Sense, did not negatively impact differentiation of labeled hMSC into osteogenic or adipogenic lineages.[19] NMR revealed the cellular loading of ^19^F varied between experiments and cell types within the range of 8.2x10^10^ to 2.4x10^11^ atoms.

### Quantification of ^19^F in cell samples

**Fig 2:**
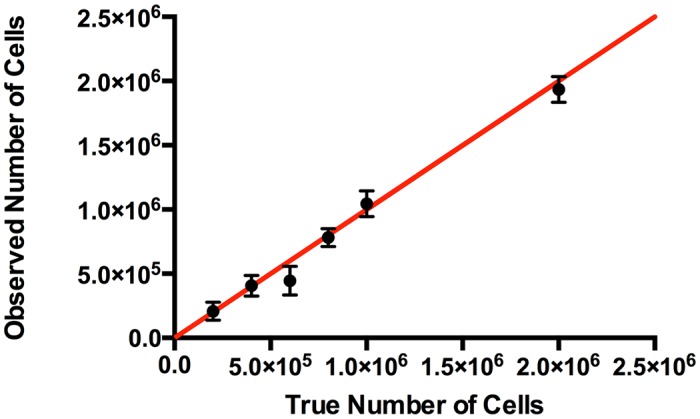
*In vitro* validation of ^19^F-MRI quantification accuracy. Quantification was validated in a phantom study using cell pellets ranging from 2x10^5^ to 2x10^6^ MSC. Pellets were imaged three times, with the error bars representing the standard deviation between scans. The ^19^F-MRI quantification is in very strong agreement with the true number of cells, and has a Pearson correlation coefficient of 0.99. The red line represents the ideal result of a 1:1 correlation.

Quantification of the ^19^F signal was tested in vitro using Cell Sense labeled mMSC pellets. Imaging was performed at 9.4T on six cell pellets ranging from 200k to 2 million cells. Fig. 2 represents the average quantification and standard deviation from imaging the cell pellets on three different occasions. We observed a strong linear relationship between the MR quantification and the real cell number, with an R^2^ = 0.98.

### In vivo detection of ^19^F MRI signal

**Fig 3:**
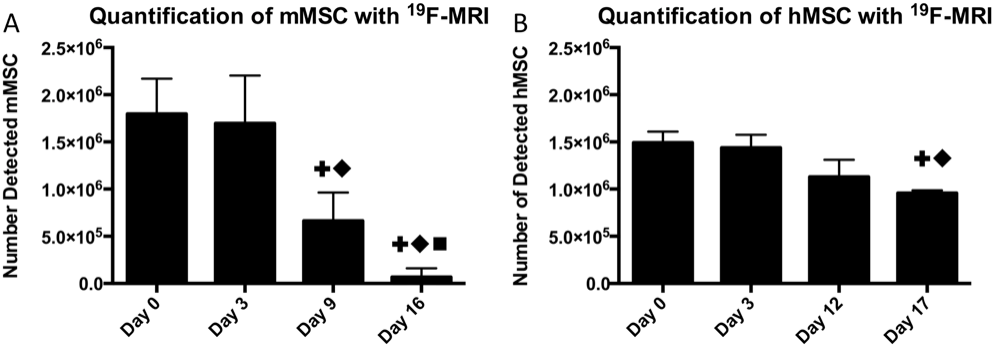
Comparison of ^19^F-labeled cell detection in two transplantation models over time. (A) Following implantation of 2x10^6^ mMSC, ^19^F-MRI was used to quantify the number of cells remaining over 16 days. By day 16, only 2/7 mice had any detectable signal remaining. A significant difference from day 0 is denoted by **+**, from day 3 by ◆, and from day 9 by ■. (B) The number of detectable cells over a similar time period following a transplant of 1.5x10^6^ hMSC. ^19^F signal was found to decrease at a slower rate, with observable signal in all mice at endpoint. Statistical significance is denoted in the same way as A.

^19^F-MRI signal was initially detectable in all mice following intramuscular injection of 2.0x10^6^ mMSC or 1.5x10^6^ hMSC. On day 0 quantification of the *in vivo* signal agreed very well with the number of implanted cells (Fig. 3). Over time the signal decreased in both models. In the immune competent model (Fig. 3A), a significant effect was observed in the decrease in ^19^F MRI signal over time [F(1.703,6.812) = 39.85, *p*<0.001]. Post hoc Tukey tests showed there was a significant difference in ^19^F signal between day 3 and day 9 (*p*<0.01), and day 9 and 16 (*p*<0.05). At 16 days post implantation only two mice had any detectable signal remaining. Signal in the immune-compromised mice (Fig. 3B) decreased more slowly [F(1.378,5.511) = 30.97, *p*<0.01], with significance from day 0 only detectable on day 17 (*p*<0.01). Furthermore, at this endpoint all immune-compromised mice still had detectable signal.

**Fig 4:**
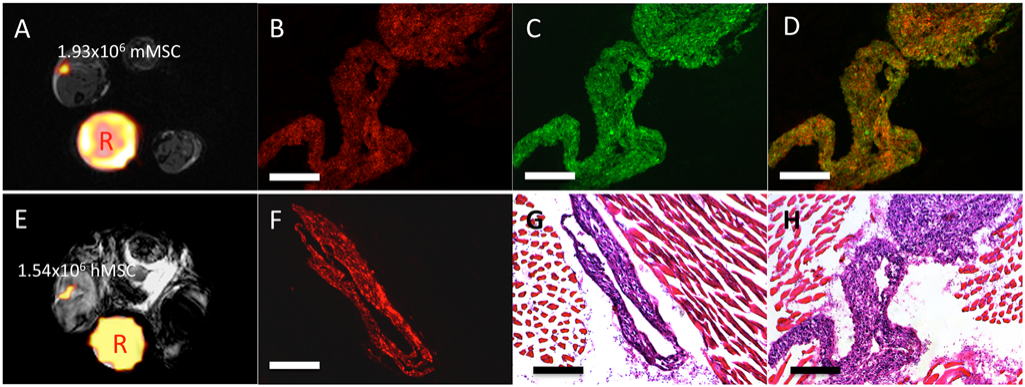
Representative Day 0 MRI, fluorescence microscopy, and histology acquired as 10x magnification from both implant models. (A, E) Representative MRI from mice receiving either 2x10^6^ mMSC or 1.5x10^6^ hMSC respectively. The day 0 *in vivo* ^19^F-MRI quantification correlates very well with the number of implanted cells. The reference tube is marked by “R”. (B) The red fluorescent fluorine agent is clearly visible in the tissue of the immune competent model, (F) as well as in the immune-compromised model. (C) Furthermore, the GFP+ mMSC are observable within the tissue section. (D) Overlaying the two fluorescent images, reveals the ^19^F agent colocalized with the GFP+ mMSC, as expected. (G, H) H&E stained tissue sections corresponding to the fluorescence microscopy clearly show the implant site of the mMSC and hMSC respectively. Scale bars in all images represent 250μm.

Representative MR image data, fluorescence microscopy, and H&E obtained on day 0 is shown in Fig. 4. Overlays of the ^19^F MRI onto the proton image at day 0 are show in 4A and E for the immune competent and immune compromised mice respectively. Fig. 4B and F show that the red fluorescence signal from the ^19^F labeling agent can be detected on day 0 in both models. The green fluorescence associated with the GFP+ mMSC was also clearly visible at the site of their implantation on day 0 (Fig. 4C). Overlaying the two fluorescent images revealed strong co-localization, with a Pearson’s correlation coefficient of 0.80, between the red fluorescent ^19^F agent and the GFP+ mMSC in Fig. 4D. The corresponding H&E stained tissue sections reveal the location of the implant within the muscle (Fig. 4G,H).

**Fig 5:**
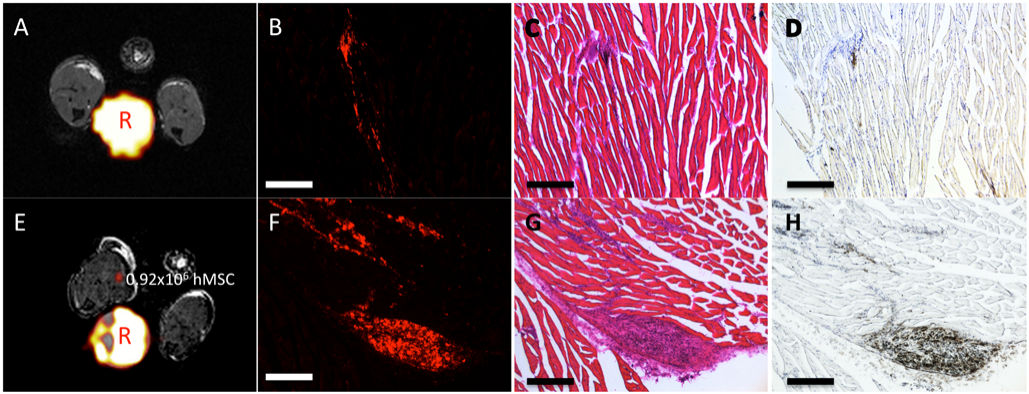
Representative endpoint MRI, fluorescence microscopy, and immunohistochemistry acquired at 10x magnification from both implant models. (A) By day 16, 5/7 immune competent mice had no ^19^F signal remaining as shown by the representative MRI. The reference tube is marked by “R”. (B) Fluorescence microscopy of the muscle tissue revealed little red fluorescence remaining in the immune competent mice. No GFP+ mMSC were detectable by fluorescence microscopy, suggesting the original mMSC are no longer present. (C) H&E staining reveals the presence of cells at the implant site which correlate well with the remaining ^19^F red fluorescence. (D) Immunohistochemistry staining of adjacent tissue sections with the anti-F4/80 antibody reveals the presence of a few macrophages at this location in the immune competent model. (E) At endpoint, all immune compromised mice had detectable ^19^F-MRI signal remaining. (F) We observed more red fluorescence from the ^19^F-label at the transplant site in the immune compromised mice. (G) Once again, H&E staining in the immune compromised model correlates well with the regions of red fluorescence. (H) Macrophage staining of the immune compromised model reveals many more F4/80 positive cells at the site of implantation. Furthermore, the fluorescence microscopy of neighboring tissue sections reveals that the red fluorescence from the ^19^F agent is in the same location as the macrophages. Once again, scale bars represent 250μm.

Fig. 5 shows representative MR image data, H&E, F4/80 immunohistochemistry and fluorescence microscopy obtained at the experimental endpoint. By day 16 post implantation no ^19^F signal from the mMSC was detectable in 5/7 of the immune competent mice. One of these mice is shown in Fig. 5A where the only ^19^F signal comes from the reference tube. In contrast, ^19^F signal was still detectable in all of the immune compromised mice at day 17, an example is shown in Fig. 5E.

Fluorescence microscopy revealed only a small area of red fluorescence from the ^19^F agent in the immune competent model (Fig. 5B) compared to the immune compromised (Fig. 5F). In addition, no GFP+ mMSC were detectable in *ex vivo* samples at endpoint. H&E staining of the same tissue sections clearly showed cells at the site of the implant, which strongly corresponded with the ^19^F agent’s red fluorescence (Fig. 5C,G). Neighboring tissue sections corresponding to high red fluorescence were stained for the presence of macrophages using F4/80. Fig. 5D shows that relatively few macrophages were detected in the immune competent model at endpoint. In contrast, the F4/80 stain revealed large numbers of macrophages (Fig. 5H) in the same region as the red fluorescence from the ^19^F agent in the immune compromised model. The number of macrophages shows very strong correspondence to the amount of remaining red fluorescence in both cases.

An important additional finding worth noting was that ^19^F signal due to the use of inhalational isoflurane anesthesia could be eliminated by using a narrow 1.5 kHz sinc excitation pulse. S1 Fig. shows that a broader 3kHz RF pulse led to substantial background ^19^F signal in the mouse body. Isoflurane becomes concentrated within fat pads during imaging[31] and these high levels of ^19^F signal can lead to false-positive detection and incorrect quantification. Isoflurane anesthesia is preferred for the ease of use and the relatively long imaging times required for ^19^F MRI. Another option is ketamine/xylazine but this requires the use of an intravenous or intraperitoneal catheter and infusion pump in order to maintain effectiveness over the time period required which can be challenging in mice.[32–34] Pentobarbital is also capable of providing anesthetic for over 2 hours[19], but its use leads to a high mortality rate in experimental animals.

## Discussion

### *In vivo* quantification of MSC

This study demonstrates the ability to use ^19^F-MRI cell tracking to detect and, most importantly, to measure the number of transplanted stem cells in vivo. Cell number cannot be quantified using other MRI cell tracking techniques. Numerous studies have reported on the challenges associated with quantification of signal loss due to iron-labeled cells.[19,20,35] This has, so far, narrowed the implementation of cell tracking for monitoring the fate of transplanted cells. In this study we show excellent correlation between the number of labeled cells implanted and the number of cells counted on day 0 by ^19^F-MRI. This capability will pave the way for MRI to be used for to confirm the delivery of therapeutic cell transplants.

The importance of accurate delivery of cells to a target tissue cannot be overstated. In preclinical investigations often stem cells will be transplanted then a set time allowed to lapse before the transplanted tissue is removed and processed for microscopy, to determine whether stem cells remain in the tissue. In many cases only a very small sample of the transplanted tissue is evaluated. In a previous study we used MRI to track iron-labeled MSC in a mouse model of spinal cord injury[36]. Our in vivo imaging revealed that the challenging intrathecal injections of MSC were imprecise approximately 25% of the time; injected cells were often being deposited in tissue above the cord or leaking out of the cord. The histological assessment in this study involved the analysis of 1cm of cord tissue on either side of the transplant site, six weeks after transplantation. Without MRI, a negative observation of MSC by microscopy would have been taken as failure to engraft rather than due to a missed injection. Injection confirmation with ^19^F MRI would have the additional advantage of determining how many cells were properly injected and remain at the site.

In a first-in-man study, MRI and scintigraphy were used to assess the success of intranodal injections consisting of a 1:1 mixture of iron- or indium-labeled dendritic cell (DC) for cancer therapy in melanoma patients.[37] Despite these cell injections being performed under ultrasound guidance the MRI of iron-labeled DC revealed that in 3/8 cases DC were injected near, but not in, the target lymph nodes.[37] These findings make it clear that the outcome of this cell therapy on these patients would not be properly evaluated without knowledge of proper delivery of DC to the nodes. For DC therapy knowledge of the number of cells delivered to a lymph node is especially critical since DC migration to nodes correlates with effective stimulation of T cells. ^19^F-MRI therefore presents a promising solution to the clinical problem of verifying both the location and number of cells within the region of interest immediately following administration of treatment.

In these experiments the hMSC and the mMSC were both capable of taking up sufficient ^19^F label for in vivo detection, without decreasing viability below 80%. We observed some variation in the number of ^19^F atoms loaded per cell between experiments, although the average loading was not significantly different for mouse versus human MSC. The cell size is one of the more important factors influencing the labeling with Cell Sense and mouse and human MSC are approximately 30 microns in diameter. The fact that it is possible to obtain robust measurements of the number of cells detected by MRI, despite inter-experiment variation in the number of ^19^F atoms per cell, is another positive feature of this type of cell tracking. Variation in ^19^F/cell is not a confounding factor in MRI quantification since the unique cellular loading is determined for each transplantation. This is particularly important when considering clinical translation, since ^19^F uptake is expected to vary between cell donors.

### Monitoring the fate of MSC over time

This study also revealed some interesting information about tracking cell fate over time with ^19^F MRI. We studied the fate of MSC in two different models: mMSC implanted into healthy wild type C57Bl/6 mice (immune competent model) and hMSC implanted into healthy nude mice (immune compromised model). The MSC underwent clearance from the sites of implantation in muscle at different rates, which is not unexpected since the two different mouse strains have very different immune systems.

In the immune competent model, 5/7 mice had no MRI detectable ^19^F signal remaining at the site of injection by the endpoint. The other 2 mice had just 4% and 10% of the original signal remaining. There were no GFP+ cells detected by fluorescence microscopy but there was some red fluorescence, indicating that some of the ^19^F label persisted at the site but that it was not within GFP+ mMSC. The small amount of residual red fluorescent signal corresponded with F4/80 staining suggesting that some transfer of ^19^F label from mMSC to macrophages has occurred. The decline in the signal is likely the result of a number of different things happening at the transplant site. First, a large number of MSC will die early after their direct transplantation into a tissue. Second, the label may be diluted by MSC proliferation, or degradation of the label within MSC. Third, MSC may have migrated away from the implant site; although we did not detect ^19^F signal in other nearby locations.

The fact that for 5/7 mice no MRI signal was detected, even though red fluorescence was still observed in the tissues (as for the example shown in Fig. 5A-D) is most likely because the number of cells containing the red fluorescent ^19^F label is very small and this small amount of ^19^F signal is below the *in vivo* detection limits of the MRI protocols used in this study.

In the immune compromised model the ^19^F signal persisted in all mice until the experimental endpoint. Much more red fluorescence was observed in these tissues at the implant site and this corresponded well with F4/80 staining, again suggesting that the persistent ^19^F signal was related to transfer of label from hMSC to macrophages. A limitation of this study was that our hMSC were not also GFP+. This would have allowed us to say with more confidence that the ^19^F label was associated with macrophages and not the implanted hMSC. Clearance of label and macrophages may have been slower in these mice because of the inhibited immune system and lack of rejection response.

Our observation of bystander cell uptake of ^19^F cell label is supported by a study by Boehm-Sturm et al.[38] In their study in vivo imaging of the location, density, and survival of neural stem cells implanted in the brain in a stroke model was performed using ^19^F MRI in combination with bioluminescence imaging. The signal from ^19^F labeled stem cells persisted for more than 4 weeks after implantation while, over the same time period, the bioluminescence declined, indicating stem cell death. Immunohistochemistry staining also revealed the presence of microglia/macrophages at the site of implantation.

Terrovitis et al. looked at the retention of iron labeled stem cells implanted into immune competent rats.[20] Either rat or human cardiac-derived stem cells were injected intramyocardially. In both cases MRI signal loss due to iron was detected for 3 weeks post cell injection and correlated with the presence of iron containing macrophages in histology. Although the area of signal void decreased over time substantial signal void persisted at the injection site in all mice. Since proton MRI is sensitive to even small numbers of iron-labeled cells this form of cell tracking is most susceptible to the misinterpretation of cell fate. Previous studies performed in our lab using iron labeled syngeneic MSC also revealed the persistence of an iron signal void past 21 days in immune competent mice.[14]

## Conclusion

In summary, ^19^F MRI can be used to provide immediate assessment of implanted cells with excellent correlation between implanted cell number and *in vivo* quantification. Over time, as the cells are cleared from the transplantation site, transfer of the ^19^F label to bystander cells may confuse interpretation of the change in ^19^F signal. With the first-in-man studies of ^19^F MRI recently completed, this result will be particularly relevant when translating this technique into the clinic.[24]

This work was supported by a research grant from the Ontario Institute for Cancer Research, Smarter Imaging Program. J.M.G. is jointly funded by the Translational Breast Cancer Research Unit and the Cancer Research and Technology Transfer training program. The funders had no role in study design, data collection and analysis, decision to publish, or preparation of the manuscript.

## Supporting Information

S1 Fig Influence of excitation pulse width and shape on isoflurane detection. (A) Strong isoflurane signal (red arrow) is detectable following accumulation in the fat pads of mice after excitation with the standard Gaussian filtered sinc pulse. This signal is affected by a chemical shift from the fat pad (blue arrow), the fluorine signal in the reference tube experiences does not shift. By applying a Gaussian filter to the sinc a more rectangular waveform is achieved after Fourier transform, but at the cost of broadening the pulse in frequency space. (C) The mouse was then scanned with a non-filtered sinc pulse. Fourier transform of this pulse produces a narrower excitation that did not excite isoflurane ^19^F atoms, preventing background signal. Both images have been windowed to the same level, and brightened to show the noise distribution. (B) The filtered pulse shape in time space is shown, with a width of 0.66ms. (D) The non-filtered sinc pulse width is much broader with a FWHM of 1.32ms in time space. This produces a narrower pulse in frequency space, preventing the excitation of isoflurane signal.
(TIF)

## References

[1] Chagastelles PC, Nardi NB, Camassola M. Biology and applications of mesenchymal stem cells. Sci Prog. 2010;93: 113–127. Available: http://www.ingentaconnect.com/content/stl/sciprg/2010/00000093/00000002/art00001 20681317 10.3184/003685010X12708175591515 PMC10365478

[2] Meirelles L da S, Nardi NB. Methodology, biology and clinical applications of mesenchymal stem cells. Front Biosci. 2009;14: 4281–4298. 19273350 10.2741/3528

[3] Pittenger MF. Multilineage Potential of Adult Human Mesenchymal Stem Cells. Science (80-). 1999;284: 143–147. 10102814 10.1126/science.284.5411.143

[4] Keating A. Mesenchymal stromal cells: new directions. Cell Stem Cell. 2012;10: 709–16. 10.1016/j.stem.2012.05.015 22704511

[5] Nauta AJ, Westerhuis G, Kruisselbrink AB, Lurvink EGa, Willemze R, Fibbe WE. Donor-derived mesenchymal stem cells are immunogenic in an allogeneic host and stimulate donor graft rejection in a nonmyeloablative setting. Blood. 2006;108: 2114–20. 16690970 10.1182/blood-2005-11-011650 PMC1895546

[6] Le Blanc K, Ringdén O. Immunomodulation by mesenchymal stem cells and clinical experience. J Intern Med. 2007;262: 509–25. 17949362 10.1111/j.1365-2796.2007.01844.x

[7] Caplan AI. Mesenchymal stem cells. J Orthop Res. 1991;9: 641–50. 1870029 10.1002/jor.1100090504

[8] Psaltis P, Zannettino A. Concise review: mesenchymal stromal cells: potential for cardiovascular repair. Stem Cells. 2008; 2201–2210. 10.1634/stemcells.2008-0428 18599808

[9] Addicott B, Willman M, Rodriguez J, Padgett K, Han D, Berman D, et al Mesenchymal stem cell labeling and in vitro MR characterization at 1.5 T of new SPIO contrast agent: Molday ION Rhodamine-B. Contrast Media Mol Imaging. 2010;6: 7–18. 10.1002/cmmi.396 20690161 PMC4410881

[10] Hill JM, Dick AJ, Raman VK, Richard B, Yu Z, Hinds KA, et al Serial cardiac magnetic resonance imaging of injected mesenchymal stem cells. 2006;108: 1009–1014. 12912822 10.1161/01.CIR.0000084537.66419.7A PMC1490325

[11] Xu C, Miranda-Nieves D, Ankrum Ja, Matthiesen ME, Phillips Ja, Roes I, et al Tracking mesenchymal stem cells with iron oxide nanoparticle loaded poly(lactide-co-glycolide) microparticles. Nano Lett. 2012;12: 4131–9. 10.1021/nl301658q 22769232 PMC3552518

[12] Reddy AM, Kwak BK, Shim HJ, Ahn C, Lee HS, Suh YJ, et al In vivo tracking of mesenchymal stem cells labeled with a novel chitosan-coated superparamagnetic iron oxide nanoparticles using 3.0T MRI. J Korean Med Sci. 2010;25: 211–9. 10.3346/jkms.2010.25.2.211 20119572 PMC2811286

[13] Li X-X, Li K-A, Qin J-B, Ye K-C, Yang X-R, Li W-M, et al In vivo MRI tracking of iron oxide nanoparticle-labeled human mesenchymal stem cells in limb ischemia. Int J Nanomedicine. 2013;8: 1063–73. 10.2147/IJN.S42578 23515426 PMC3598527

[14] Noad J, Gonzalez-Lara LE, Broughton HC, McFadden C, Chen Y, Hess D a, et al MRI tracking of transplanted iron-labeled mesenchymal stromal cells in an immune-compromised mouse model of critical limb ischemia. NMR Biomed. 2012; 10.1002/nbm.2884 23165968

[15] Mathiasen AB, Hansen L, Friis T, Thomsen C, Bhakoo K, Kastrup J. Optimal Labeling Dose, Labeling Time, and Magnetic Resonance Imaging Detection Limits of Ultrasmall Superparamagnetic Iron-Oxide Nanoparticle Labeled Mesenchymal Stromal Cells. Stem Cells Int. 2013;2013 Available: http://www.hindawi.com/journals/sci/2013/353105/abs/ 10.1155/2013/353105 PMC3614076 23577035

[16] Heyn C, Bowen CV, Rutt BK, Foster PJ. Detection threshold of single SPIO-labeled cells with FIESTA. Magn Reson Med. 2005;53: 312–20. 15678551 10.1002/mrm.20356

[17] Shapiro EM, Sharer K, Skrtic S, Koretsky AP. In vivo detection of single cells by MRI. Magn Reson Med. 2006;55: 242–9. 16416426 10.1002/mrm.20718

[18] Bonetto F, Srinivas M, Heerschap A, Mailliard R, Ahrens ET, Figdor CG, et al A novel (19)F agent for detection and quantification of human dendritic cells using magnetic resonance imaging. Int J Cancer. 2011;129: 365–73. 10.1002/ijc.25672 20839261 PMC3085097

[19] Ribot EJ, Gaudet JM, Chen Y, Gilbert KM, Foster PJ. In vivo MR detection of fluorine-labeled human MSC using the bSSFP sequence. Int J Nanomedicine. 2014;9: 1731–9. 10.2147/IJN.S59127 24748787 PMC3986292

[20] Terrovitis J, Stuber M, Youssef A, Preece S, Leppo M, Kizana E, et al Magnetic resonance imaging overestimates ferumoxide-labeled stem cell survival after transplantation in the heart. Circulation. 2008;117: 1555–62. 10.1161/CIRCULATIONAHA.107.732073 18332264

[21] Ahrens ET, Flores R, Xu H, Morel PA. In vivo imaging platform for tracking immunotherapeutic cells. Nat Biotechnol. 2005;23: 983–7. 16041364 10.1038/nbt1121

[22] Morawski AM, Winter PM, Yu X, Fuhrhop RW, Scott MJ, Hockett F, et al Quantitative “magnetic resonance immunohistochemistry” with ligand-targeted (19)F nanoparticles. Magn Reson Med. 2004;52: 1255–62. 15562481 10.1002/mrm.20287

[23] Ruiz-Cabello J, Walczak P, Kedziorek Da, Chacko VP, Schmieder AH, Wickline S a, et al In vivo “hot spot” MR imaging of neural stem cells using fluorinated nanoparticles. Magn Reson Med. 2008;60: 1506–11. 10.1002/mrm.21783 19025893 PMC2597664

[24] Ahrens ET, Helfer BM, O’Hanlon CF, Schirda C. Clinical cell therapy imaging using a perfluorocarbon tracer and fluorine-19 MRI. Magn Reson Med. 2014;00: n/a–n/a. 10.1002/mrm.25454 PMC4253123 25241945

[25] Dayan V, Yannarelli G, Billia F, Filomeno P, Wang X-H, Davies JE, et al Mesenchymal stromal cells mediate a switch to alternatively activated monocytes/macrophages after acute myocardial infarction. Basic Res Cardiol. 2011;106: 1299–310. 10.1007/s00395-011-0221-9 21901289

[26] Helfer BBM, Balducci A, Nelson AD, Janjic JM, Gil RR, Kalinski P, et al Functional assessment of human dendritic cells labeled for in vivo (19)F magnetic resonance imaging cell tracking. Cytotherapy. 2010;12: 238–50. 10.3109/14653240903446902 20053146 PMC5796768

[27] Scheffler K, Lehnhardt S. Principles and applications of balanced SSFP techniques. Eur Radiol. 2003;13: 2409–18. 12928954 10.1007/s00330-003-1957-x

[28] Abràmoff MD, Hospitals I, Magalhães PJ, Abràmoff M. Image Processing with ImageJ. Biophotonics Int. 2004;

[29] Ca Schneider, Rasband WS, Eliceiri KW. NIH Image to ImageJ: 25 years of image analysis. Nat Methods. Nature Publishing Group; 2012;9: 671–675. 22930834 10.1038/nmeth.2089 PMC5554542

[30] Srinivas M, Morel PA, Ernst La, Laidlaw DH, Ahrens ET. Fluorine-19 MRI for visualization and quantification of cell migration in a diabetes model. Magn Reson Med. 2007;58: 725–34. 17899609 10.1002/mrm.21352

[31] Becker DE, Rosenberg M. Nitrous oxide and the inhalation anesthetics. Anesth Prog. 2008;55: 124–30; quiz 131–2. 10.2344/0003-3006-55.4.124 19108597 PMC2614651

[32] Srinivas M, Turner MS, Morel Pa, Janjic JM, Laidlaw DH, Ahrens ET. In vivo cytometry of antigen-specific T cells using 19F MRI. Magn Reson Med. 2009;62: 747–753. 10.1002/mrm.22063 19585593 PMC2763624

[33] Janjic JM, Srinivas M, Kadayakkara DKK, Ahrens ET. Self-delivering Nanoemulsions for Dual Fluorine-19 MRI and Fluorescence Detection. 2008; 2832–2841. 10.1021/ja077388j 18266363

[34] Partlow KC, Chen J, Brant Ja, Neubauer AM, Meyerrose TE, Creer MH, et al 19F magnetic resonance imaging for stem/progenitor cell tracking with multiple unique perfluorocarbon nanobeacons. FASEB J. 2007;21: 1647–54. 17284484 10.1096/fj.06-6505com

[35] Frangioni JV, Hajjar RJ. In vivo tracking of stem cells for clinical trials in cardiovascular disease. Circulation. 2004;110: 3378–83. 15557385 10.1161/01.CIR.0000149840.46523.FC

[36] Gonzalez-Lara LE, Xu X, Hofstetrova K, Pniak A, Chen Y, McFadden CD, et al The use of cellular magnetic resonance imaging to track the fate of iron-labeled multipotent stromal cells after direct transplantation in a mouse model of spinal cord injury. Mol Imaging Biol. 2011;13: 702–11. 10.1007/s11307-010-0393-y 20686855

[37] De Vries IJM, Lesterhuis WJ, Barentsz JO, Verdijk P, van Krieken JH, Boerman OC, et al Magnetic resonance tracking of dendritic cells in melanoma patients for monitoring of cellular therapy. Nat Biotechnol. 2005;23: 1407–13. 16258544 10.1038/nbt1154

[38] Boehm-Sturm P, Aswendt M, Minassian A, Michalk S, Mengler L, Adamczak J, et al A multi-modality platform to image stem cell graft survival in the naïve and stroke-damaged mouse brain. Biomaterials. Elsevier Ltd; 2014;35: 2218–26. 10.1016/j.biomaterials.2013.11.085 24355489

