# Supplementary figures and images for: Tracking the Fate of Stem Cell Implants with Fluorine-19 MRI
Source: PLoS One. 2015 Mar 13;10(3):e0118544. doi: 10.1371/journal.pone.0118544 (PMC4358825; doi:10.1371/journal.pone.0118544)

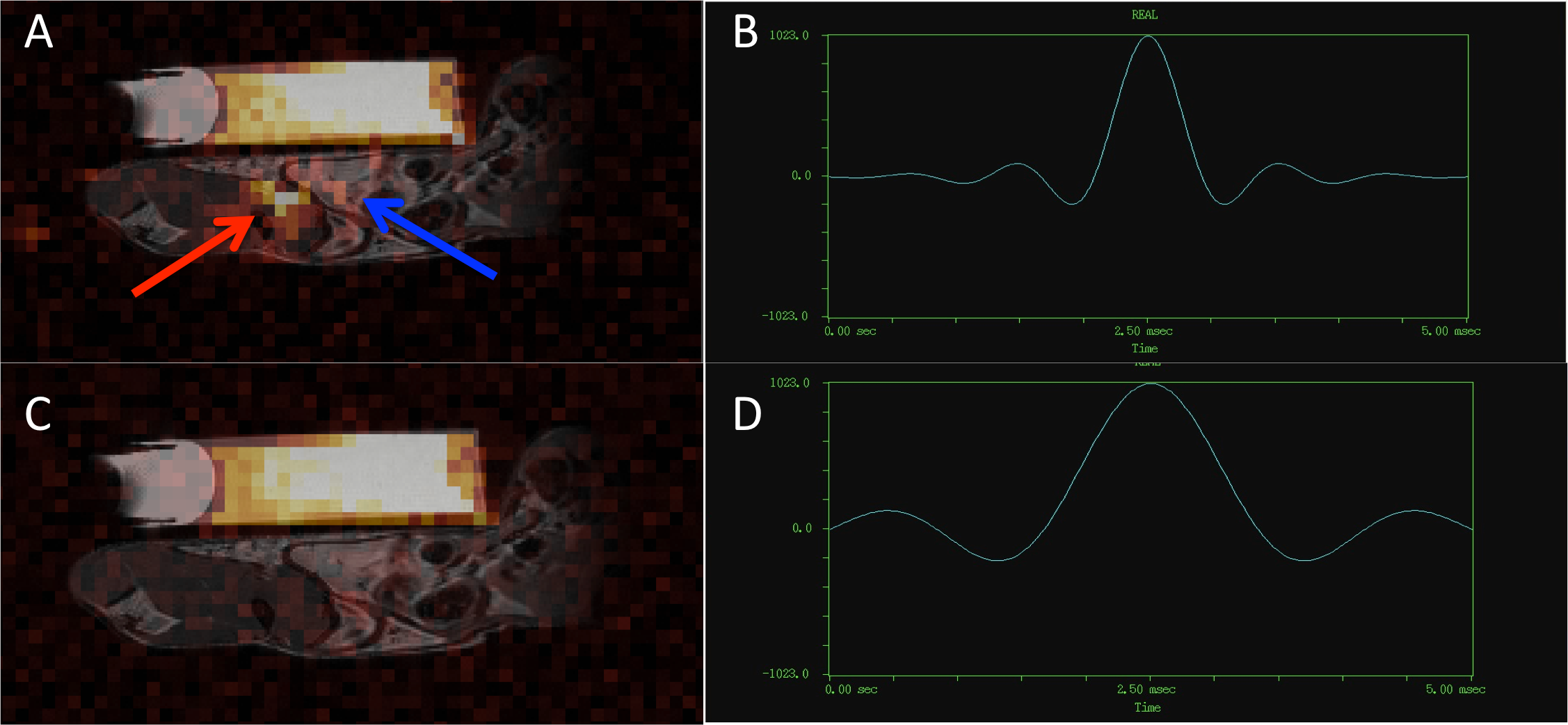

Supplement: S1 Fig — (A) Strong isoflurane signal (red arrow) is detectable following accumulation in the fat pads of mice after excitation with the standard Gaussian filtered sinc pulse. This signal is affected by a chemical shift from the fat pad (blue arrow), the fluorine signal in the reference tube experiences does not shift. By applying a Gaussian filter to the sinc a more rectangular waveform is achieved after Fourier transform, but at the cost of broadening the pulse in frequency space. (C) The mouse was then scanned with a non-filtered sinc pulse. Fourier transform of this pulse produces a narrower excitation that did not excite isoflurane 19F atoms, preventing background signal. Both images have been windowed to the same level, and brightened to show the noise distribution. (B) The filtered pulse shape in time space is shown, with a width of 0.66ms. (D) The non-filtered sinc pulse width is much broader with a FWHM of 1.32ms in time space. This produces a narrower pulse in frequency space, preventing the excitation of isoflurane signal. (TIF) [file pone.0118544.s001.tif]
